# Dosimetric evaluation of respiratory gated volumetric modulated arc therapy for lung stereotactic body radiation therapy using 3D printing technology

**DOI:** 10.1371/journal.pone.0208685

**Published:** 2018-12-26

**Authors:** KyoungJun Yoon, Chiyoung Jeong, Sung-woo Kim, Byungchul Cho, Jungwon Kwak, Su Ssan Kim, Si Yeol Song, Eun Kyung Choi, SeungDo Ahn, Sang-Wook Lee

**Affiliations:** Department of Radiation Oncology, Asan Medical Center, University of Ulsan College of Medicine, Seoul, Republic of Korea; North Shore Long Island Jewish Health System, UNITED STATES

## Abstract

**Purpose:**

This study aimed to evaluate the dosimetric accuracy of respiratory gated volumetric modulated arc therapy (VMAT) for lung stereotactic body radiation therapy (SBRT) under simulation conditions similar to the actual clinical situation using patient-specific lung phantoms and realistic target movements.

**Methods:**

Six heterogeneous lung phantoms were fabricated using a 3D-printer (3DISON, ROKIT, Seoul, Korea) to be dosimetrically equivalent to actual target regions of lung SBRT cases treated via gated VMAT. They were designed to move realistically via a motion device (QUASAR, Modus Medical Devices, Canada). Using the lung phantoms and a homogeneous phantom (model 500–3315, Modus Medical Devices), film dosimetry was performed with and without respiratory gating for VMAT delivery (TrueBeam STx; Varian Medical Systems, Palo Alto, CA, USA). The measured results were analyzed with the gamma passing rates (GPRs) of 2%/1 mm criteria, by comparing with the calculated dose via the AXB and AAA algorithms of the Eclipse Treatment Planning System (version 10.0.28; Varian Medical Systems).

**Results:**

GPRs were greater than the acceptance criteria 80% for all film measurements with the stationary and homogeneous phantoms in conventional QAs. Regardless of the heterogeneity of phantoms, there were no significant differences (*p* > 0.05) in GPRs obtained with and without target motions; the statistical significance (*p* = 0.031) was presented between both algorithms under the utilization of heterogeneous phantoms.

**Conclusions:**

Dosimetric verification with heterogeneous patient-specific lung phantoms could be successfully implemented as the evaluation method for gated VMAT delivery. In addition, it could be dosimetrically confirmed that the AXB algorithm improved the dose calculation accuracy under patient-specific simulations using 3D printed lung phantoms.

## Introduction

Respiratory gated volumetric modulated arc therapy (VMAT) has been reported to sustain tumor dose conformity and normal organ sparing despite the significant target motion induced by the patient’s respiration during beam delivery [1–3]. Recently, gating technology in VMAT has become clinically available to improve the dosimetric efficacy of stereotactic body radiotherapy (SBRT) to respiratory moving targets.

Currently, respiratory gated VMAT is one of the most complicated techniques used in radiation therapy. During beam delivery, the dose rate, gantry rotation speed, and multi-leaf collimator (MLC) leaf motions are modulated in order to achieve the desired dose distribution. Also, gated VMAT is intentionally interrupted to synchronize with the patient’s respiratory cycle using a respiratory patient monitoring (RPM, Varian Medical Systems, Palo Alto, CA, USA) system [4].

Common sense suggests that end-to-end verification procedures in a realistic simulation should be employed in the clinical planning phase before the implementation of such a complicated beam delivery technique. Two factors should be considered for the realistic simulation of gated VMAT delivery in lung SBRT. One is the patient-specific heterogeneity near the target and the other is the target motion induced by the patient-specific respiratory pattern.

Many published reports have addressed the dose calculation errors of lung SBRT, in which the electronic disequilibrium due to lung heterogeneities in the small field irradiations should be taken into account [5,6]. AcurosXB (AXB), which is a Class-C dose calculation algorithm based on a Linear Boltzmann Transport Equation, was developed as an alternative approach to the Monte Carlo simulation method. Under heterogeneous conditions, the AXB algorithm has been shown to produce a more valid and accurate dose calculation than the Anisotropic Analytical Algorithm (AAA) or the Class-B model-based dose calculation algorithm [7].

Unstable breathing patterns and asynchronous target motions to respiratory phases might significantly affect the accuracy of dose delivery in gated radiotherapy [8,9]. Several studies have suggested that respiratory-induced tumor movement should be considered in overall radiation therapy procedures such as treatment planning, image guidance, beam delivery, and QA verification [10–14].

Recently, several studies on gated VMAT for SBRT have reported using a homogeneous dynamic phantom under both static and gating conditions [1,3,13,15]. However, no study has shown the dosimetric results of gated VMAT while considering the heterogeneity and realistic target motion in lung SBRT.

Conventional phantoms in QA tasks were composed of mainly water equivalent and homogeneous material and formed as simple shapes like boxes, cylinders and spheres. For some heterogeneity conditions, slabs made of lung tissue and bone equivalent materials might be appended to the homogeneous media. However, the patient-specific implementation of actual organ tissues has been morphologically limited with such phantoms. In addition, the realistic respiratory motions have generally not been considered [1,3,4].

In this study, the dosimetric verification of gated VMAT using 3D printed patient-specific lung phantoms was performed by simulating real patients’ target movements and the heterogeneity around the targets. Also, the relative verification using a stationary homogeneous phantom as the reference of conventional QA was carried out to examine the usefulness of the heterogeneous phantom.

Using 3D printing technology, some studies have reported on the various patient-specific applications of homogeneous mediums in radiation therapy (e.g., skin bolus, compensators, and patient-specific phantoms) [16–18]. However, the patient-specific heterogeneity has been rarely implemented except of the 3D printed lung phantoms employed in this study.

Previously, using the 3D printed lung phantoms, some papers were published to confirm the accuracy of the Cyberknife Xsight Lung Tracking System [19] and to report the preliminary results of a single case [20]. However, even if the same phantoms had been partially enrolled in the previous studies, the utilization as imaging phantom and the preliminary study of 3D printing fabrication methods would be clearly distinguishable with the aim of this study. In the previous article reporting a single event, the fabrication processes of 3D printed lung phantom was mainly mentioned without any statistical analysis. For a robust conclusion on the dosimetric accuracy of gated VMAT under realistic conditions, patient specificity should be considered by enrolling more cases.

## Materials and methods

### 3D-printed patient-specific moving lung phantom

A new approach was applied to produce patient-specific lung phantoms using 3D printing technology based on patients’ computed tomography (CT) data in this study. While the morphology of lung tissue is too complex to be completely implemented with generic 3D printers, the effective similarity and accuracy of physical density for dosimetric applications was mainly considered in fabricating the lung phantoms.

Six patients who had been treated with gated VMAT for lung SBRT were enrolled in this study. The patients were selected to involve various and representative conditions as much as possible for phantom fabrication. The tumor volumes, location, lung tissue volume (LTV), respiratory motion amplitudes, and target margins of the six cases are summarized in Table 1. The average amplitude of respiratory tumor motions during free breathing was 13.48 ± 3.56 mm, which was reduced to 3.08 ± 0.75 mm as residual movements within the gating window. While the average volume of the actual gross tumor volumes (GTVs) was 10.47 cm^3^ (ranging 1.9–21.5 cm^3^), the mean value of the 3D printed tumor volumes was 10.93 cm^3^ (ranging 2.1–22.1 cm^3^). The study was approved by the Institutional Review Board of Asan Medical Center, and informed consent was waived because of the retrospective nature of the study.

**Table 1:** The tumor volumes, respiratory-tumor motion, and tumor margins for six cases.

No	GTV (cc)		Location	^a^LTV (cc)	Full motion (mm)	Margin (mm)	
	Patient	Phantom				^c^ITV	^d^PTV
**P1**	1.9	2.1	LUL	290.0	7.2	2.6	5.0
**P2**	4.4	4.6	RUL	305.7	12.4	1.8	5.0
**P3**	8.3	9.3	RUL	251.5	13.5	3.8	5.0
**P4**	17.9	18.6	LLL	186.9	14.6	3.6	5.0
**P5**	8.8	8.9	RUL	134.5	17.6	3.2	5.0
**P6**	21.5	22.1	RLL	226.1	15.6	3.5	5.0
**Mean**	10.47	10.93		232.45	13.48	3.08	5.0
^**b**^**SD**	7.68	7.85		64.42	3.56	0.75	

Abbreviations: GTV, gross tumor volume; ITV, internal target volume; PTV, planning target volume; LLL, left lower lobe; LUL, left upper lobe; RLL, right lower lobe; RUL, right upper lobe.

^**a**^LTV, lung tissue volume with a mesh structure with a 2 mm air gap except for the tumor site in the 3D printed patient lung phantom

^**b**^SD, standard deviation

^**c**^ITV = GTV + gating phase window (30–70%)

^**d**^PTV = ITV + 5 mm isotropic margin

**Fig 1:**
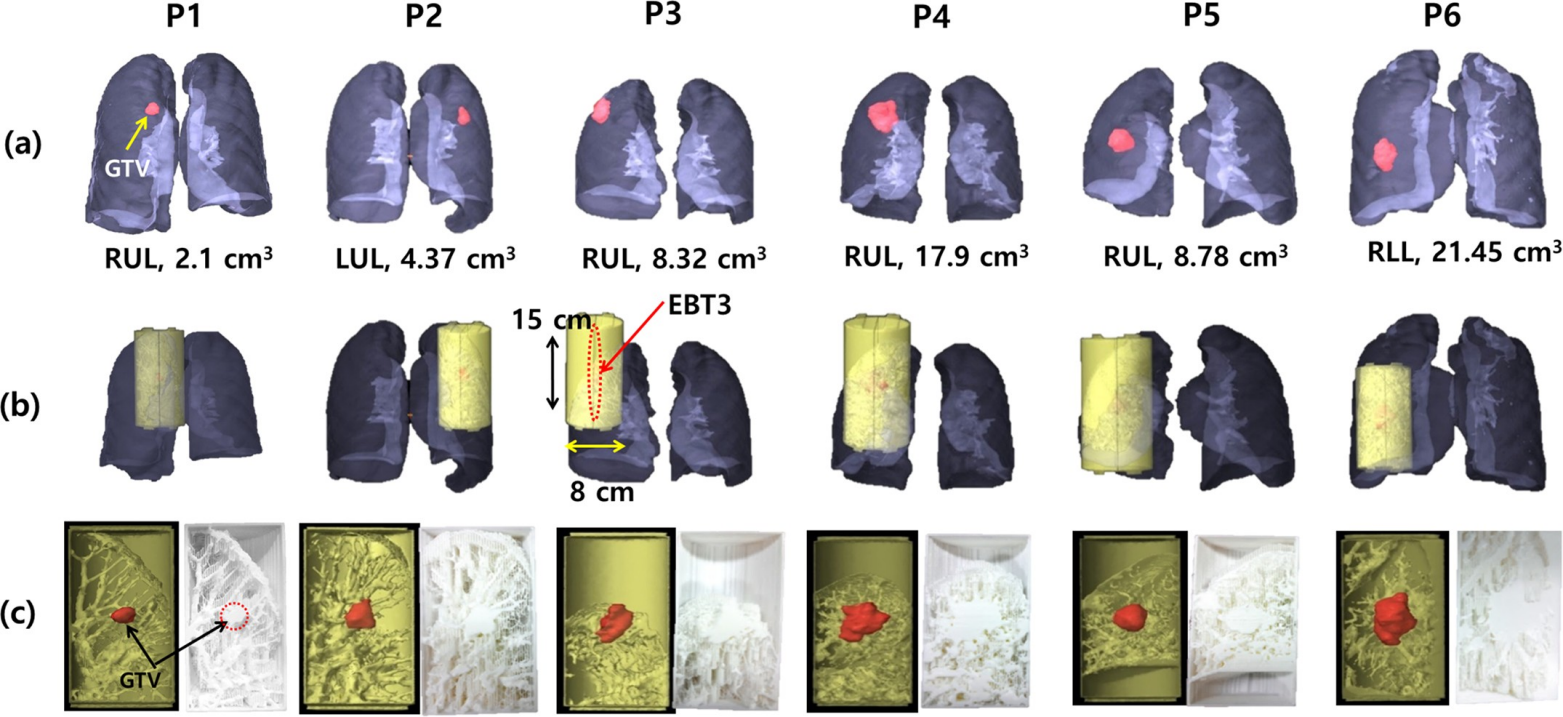
3D printed lung phantom. (a) The GTV volume and location of the six patient cases, (b) Design of a 3D printed lung phantom, (c) Rendering image (left) and fabricated 3D-printed lung phantom (right).

As shown in Fig 1(A), the lung phantoms were designed on the basis of the end-of-exhale phase image extracted from a 4D-CT (LightSpeed RT16, GE Healthcare, USA) data set. The phantom insert was designed to be cylindrical with a diameter of 8 cm and length 15 cm to fit into the respiratory motion device (QUASAR, Modus Medical Devices, Canada), while the center of each GTV was placed at the middle of the phantom. The cylindrical insert could be split in half in order to embed EBT3 film (Gafchromic, International Specialty Products, Wayne, NJ) for 2D dose measurements.

A radiodensity of -700 Hounsfield units (HU) observed in the end-of-exhale phase images was set as a threshold to distinguish between high-density areas like the GTV and pulmonary veins and the low-density regions in lung tissues. In 3D printing the phantoms, the region filled with mesh structure was intended to represent the lung tissue around tumors in the phantoms. On the other hand, the fully filled regions with filament material were represented for volumes with water equivalent density in lung tissues.

Fig 1(C) shows the rendering image (left) and 3D-printed lung phantom (right) representing the tumor and surrounding lung tissue of the six lung cancer patients.

The DICOM files including segmented contours were imported to the 3D Slicer program (version 4.3.1, NIH-supported open-source platform, http://www.slicer.org) [21], in which the geometric information of contours was converted to a Standard Tessellation Language (STL) format. Using a CreatorK program (ROKIT, Seoul, Korea), the STL file was translated in G-cord format for a Fused Deposition Modeling (FDM) based 3D-printer (3DISON, ROKIT, Seoul, Korea). As 3D printing specifications for lung phantoms, 0.4 mm nozzle diameter, 1.75 mm filament diameter, 215°C nominal nozzle temperature, 0.3 mm layer thickness, and 40 mm/s printing speed were set. The filaments consisting of poly-lactic acid (PLA) with a density of 1.2 g/cm^3^ was utilized as the filling material.

**Fig 2:**
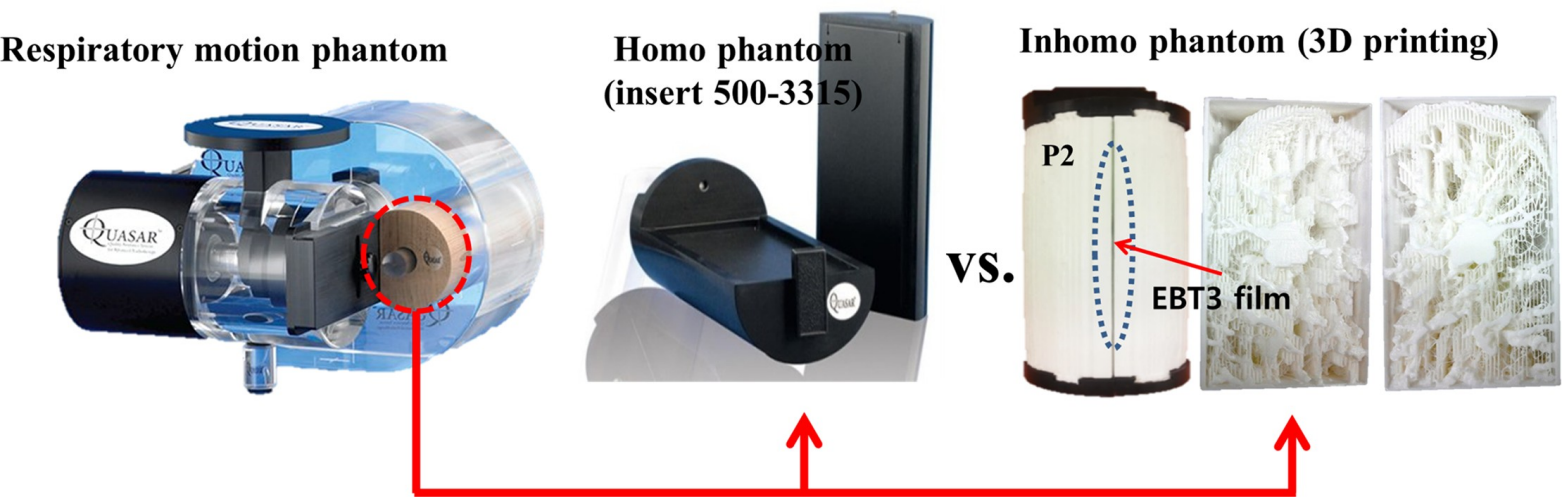
4D phantom with 3D printed lung phantom. The inserts are either a 3D printed lung phantom (inhomogeneous) or a homogeneous phantom.

As shown in Fig 2, a respiratory motion simulator consists of cylindrical inserts and Quasar respiratory moving device. A homogeneous insert module (insert model 500–3315, acrylic materials) was employed as reference phantom to clarify the dosimetric effect of the heterogeneity of patient-specific lung phantoms.

### Validation of the patient-specific phantom

**Fig 3:**
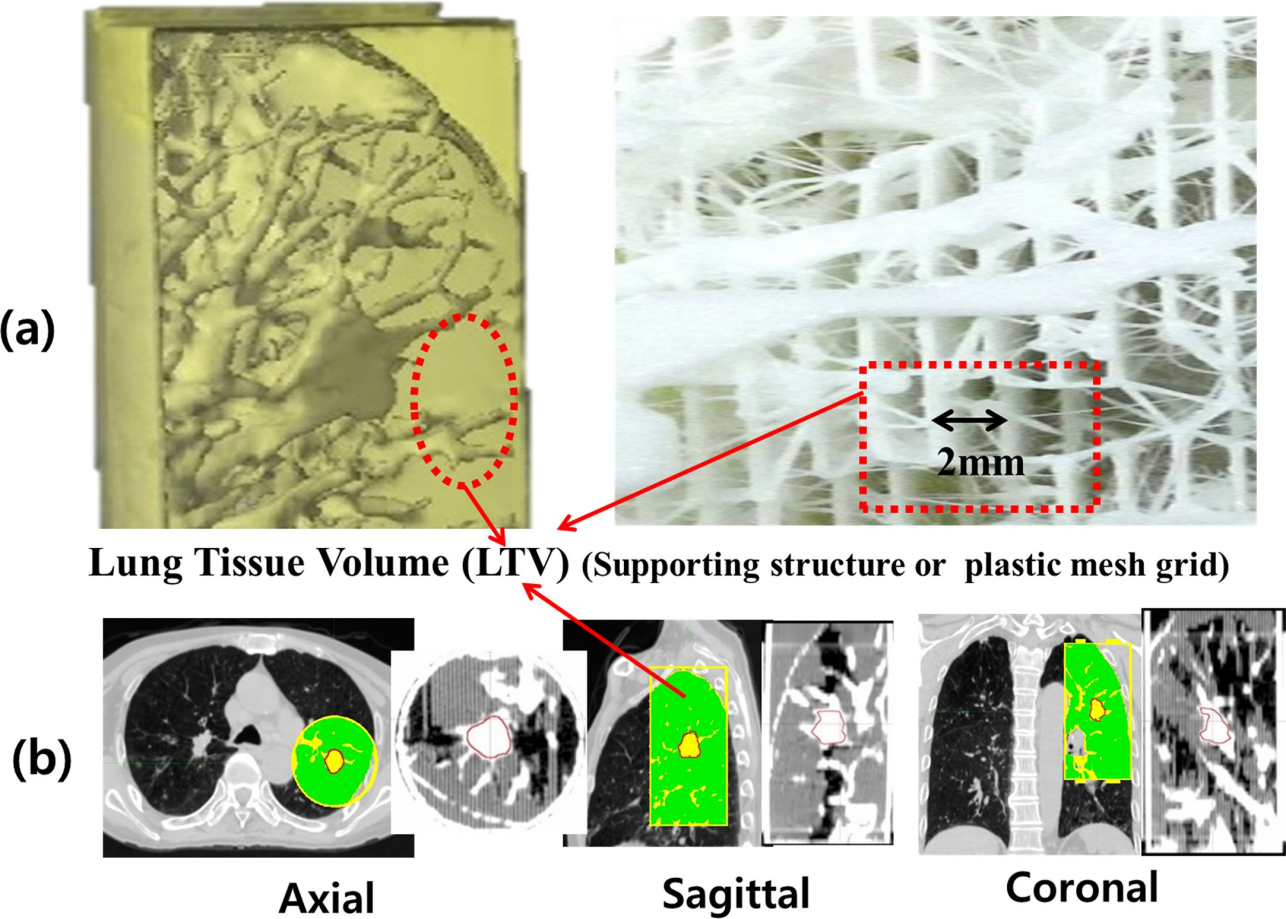
The lung tissue volume (LTV) of the phantom. (a) The LTV is filled with 0.3 mm strips with a 2 mm air gap to match the lung density. (b) The green area is the LTV in the phantom.

As shown in Fig 3(A), the lung tissue area with HU values lower than -700 was filled with mesh structure of 0.3 mm thick strips by 2 mm spacing in the 3D printing process. The LTV was defined as the mesh region inside the cylindrical phantom, presented as the green colored area in Fig 3(B). To confirm the similarity of the 3D-printed phantom to the actual lung tumor site, the average HU values of the tumor and surrounding lung tissue were compared. Additionally, the dosimetric impact of LTV was evaluated by overriding the original CT numbers of the selected area to obtain HU values. The original HU values in LTVs were manually reassigned with air equivalent values (-1000 HU), nominal lung value (-785 HU), and water equivalent values (0 HU) in the treatment planning system. The dose in the original CT was compared with the calculated dose in the corrected images using the gamma passing rate (GPR) of 3%/0 mm criteria using the OmniPro I’mRT (IBA Dosimetry, Germany) software.

### 4D-CT scan and VMAT planning

**Fig 4:**
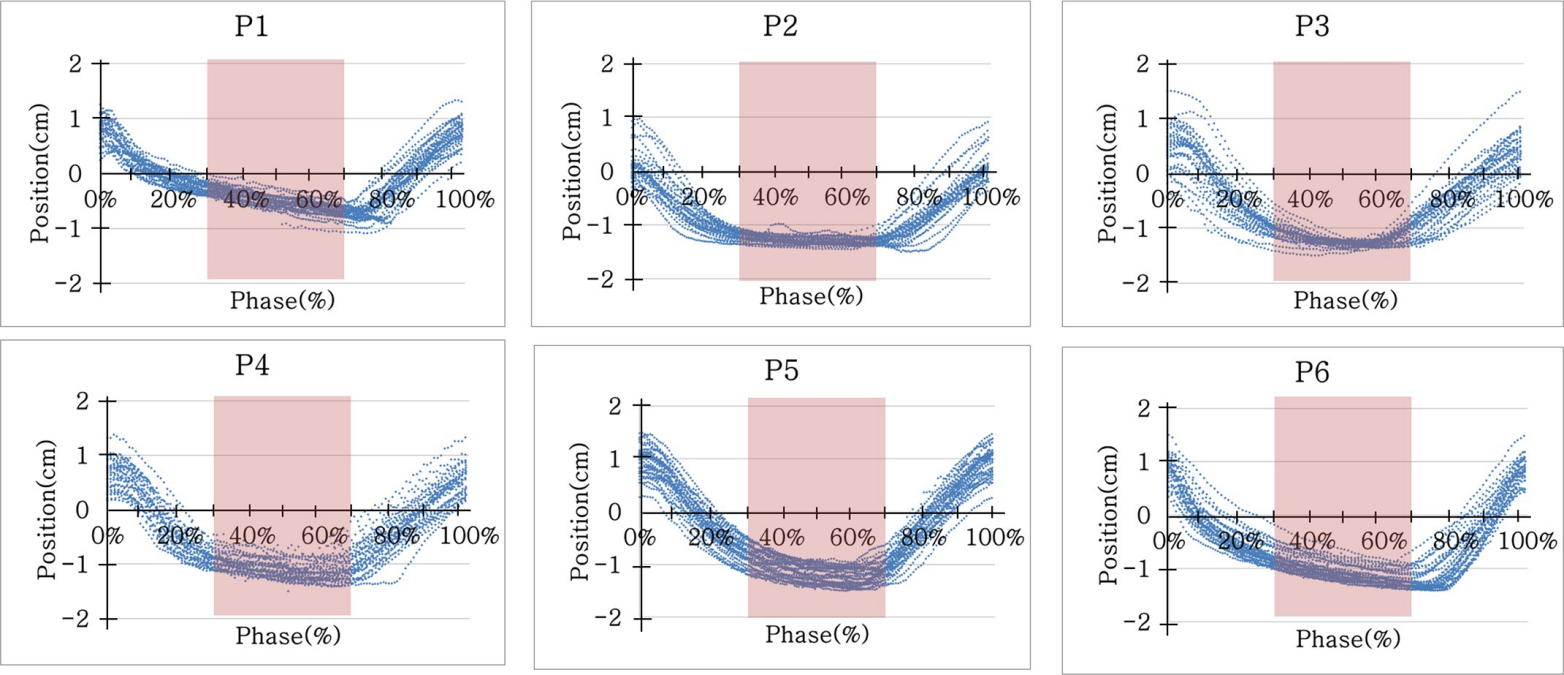
Patient-specific respiratory data. Six patient-specific breathing patterns were acquired from the Varian RPM system during planning CT scan for each patient.

The six patient-specific breathing patterns were acquired for more than 4 minutes using the RPM system during plan CT scanning, as shown in Fig 4. The acquired motion data could repeatedly be reproduced over 60 minutes using a Quasar programmable respiratory motion program.

**Fig 5:**
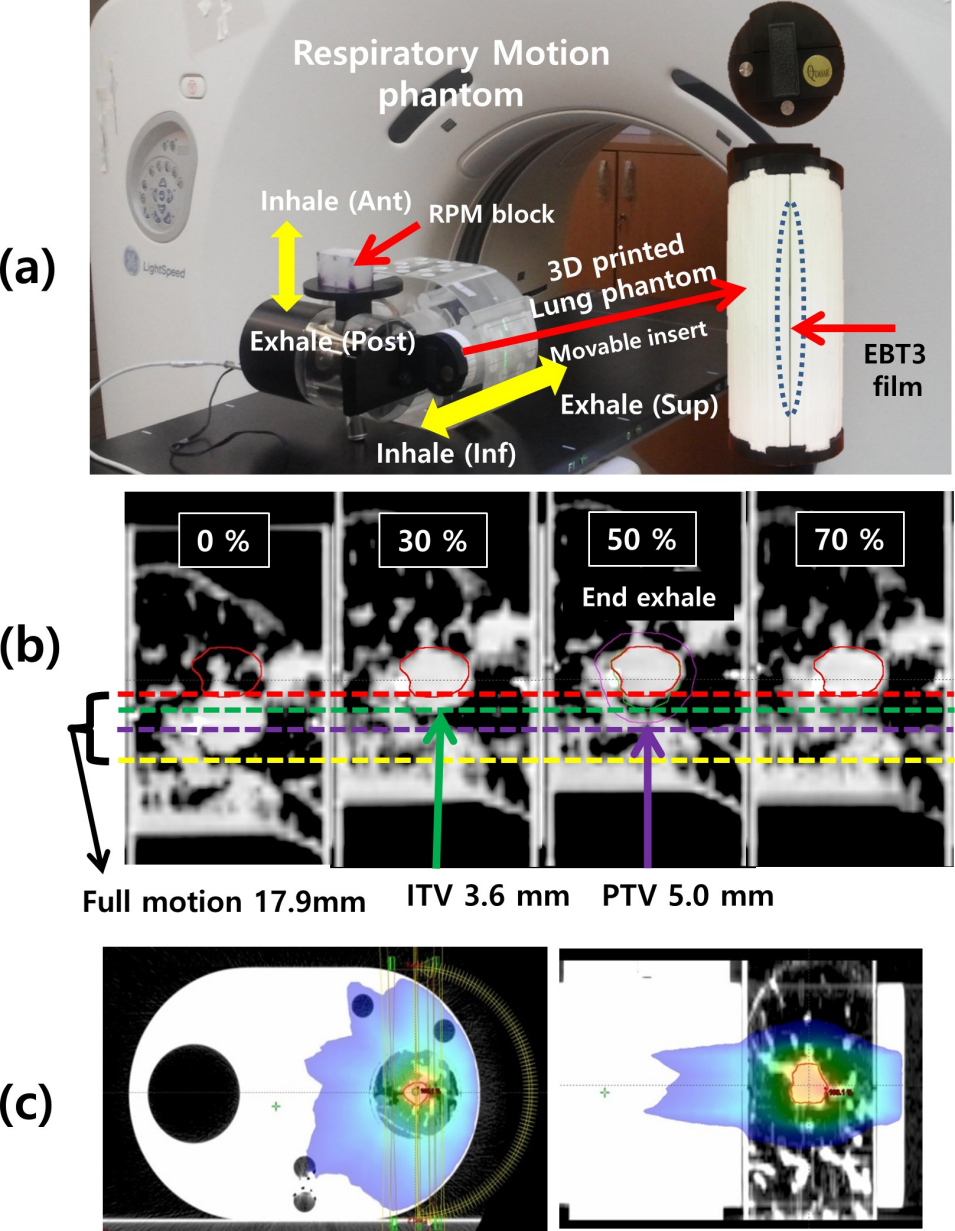
4D-CT scan of the 4D-lung phantom for case P4. (a) A respiratory motion device and the 3D-printed lung phantom insert. The EBT3 film could be placed inside phantoms. (b) 4D-CT image set of the lung phantom was sorted in phases. The images listed from left to right correspond to the breathing phases of end-inhale (0%), mid-exhale (30%), end-exhale (50%), and mid-inhale (70%), respectively. The 17.9 mm full motion was reduced to 3.6 mm residual motion in the 30–70% gating window. (c) VMAT plan dose distribution for the lung phantom at the end-exhale (50%) phase.

As shown for the fifth case in Fig 5(A), the 4D-CT image set of the patient-specific phantom was acquired with 1.25 mm slice thickness and sorted into 10 phases via the advanced workstation (AW, GE Healthcare) system. Also, the 4D-CT images for planning of the phantoms were taken with dummy EBT film insertion.

In the Eclipse TPS, the GTV was manually contoured on the end exhale-phase image of the 4D-CT set. As shown in Fig 5(B), to impose the tumor volume on the residual movement within the respiratory gating window, each internal target volume (ITV) was generated based on the maximum intensity projection image for the breath phases ranging from 30 to 70%. Finally, the 5mm isotropic extension for planning target volume (PTV) margin was applied at the ITV. In the end-exhale phase, the double arc VMAT plans for 6 phantoms were established using the Eclipse TPS version 10.0, as shown in Fig 5(C). A 6 MV photon beam with 600 MU/min dose rate was employed to deliver a 5 Gy prescription dose to the PTV. The AAA and AXB algorithms were utilized to calculate the plan doses of VMATs with 2.5 mm grid size.

### Beam delivery

The VMAT verification plans were delivered via a TrueBeam STx system (Varian Medical Systems, Palo Alto, CA) equipped with a high-definition multi-leaf collimator (HDMLC-120) and RPM system. Prior to beam delivery, the image guidance was applied similar to the actual clinic setting, using 2D fluoroscopic and 3D cone beam CT images acquired using the onboard imaging system. The dosimetric films were subjected to be exposed only under treatment beam deliveries. To compare with the conventional QA procedures, the same plans were delivered under static conditions with stationary phantoms and the gated conditions with realistic phantom motions.

### Comparison of dosimetric accuracy and statistical analysis

The gamma analysis was performed to compare the calculated plan dose with the measured film dose maps. As recommended for QA procedures of SBRT cases with HDMLC, the criteria for 2D global GPR were set to have a 2% dose difference and 1 mm distance to agreement with 10% low-dose threshold for all pixels in each 6 × 12 cm region of interest, in which 80% of GPR would be recommended as an acceptance threshold[22]. The digitized images were extracted from the irradiated film via a flat panel scanner (Epson 10000XL, NISCA Inc.) in 48 bit red-green-blue (RGB) mode at a resolution of 72 dpi (0.35 mm pixel size). Using FilmQA pro 2015 (Ashland Inc., Bridgewater, NJ) software, the images were converted to 2D dose distributions, which were compared with plan doses. By comparing between VMAT plans and corresponding film measurement, the 8 GPR sets for 6 treatment cases were generated under the combinations of conditions for the target motions, the heterogeneities of phantoms, and the dose calculation algorithms. Since each set of GPR values did not meet the regularity verification, the Wilcoxon signed rank test was performed using SPSS Statistics version 20.0 (IBM Corp., Armonk, NY, USA) to determine the statistical significance of QA results under various conditions. Statistical significance was set at *p* < 0.05.

## Results

**Fig 6:**
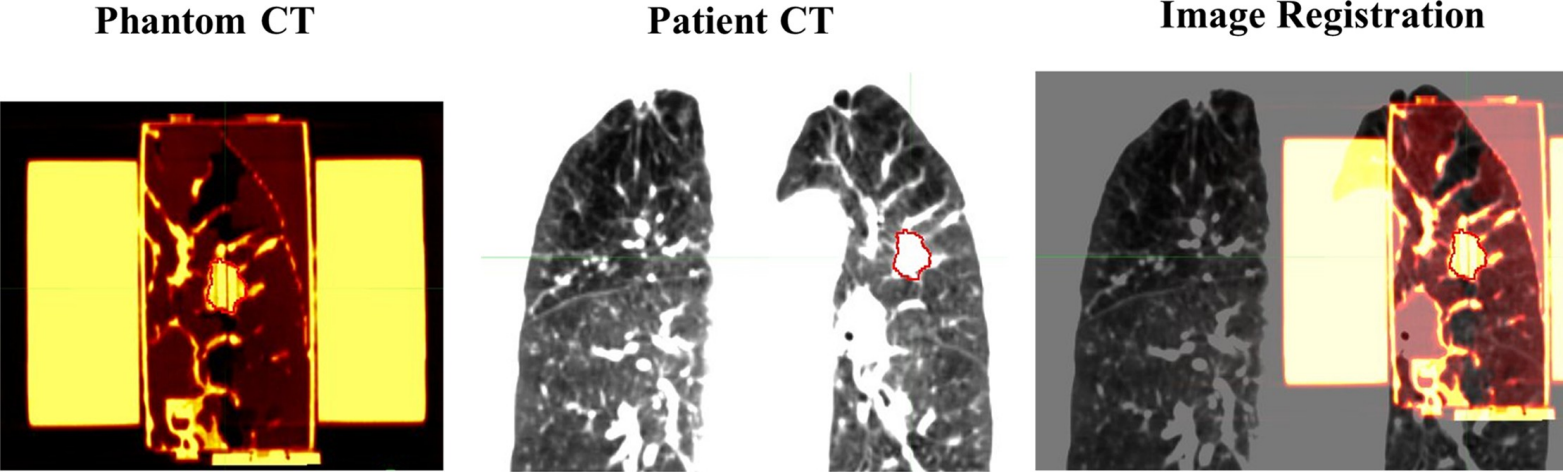
Image registration between the patient CT and lung phantom CT. Both CT image were used in the coronal plane.

**Fig 7:**
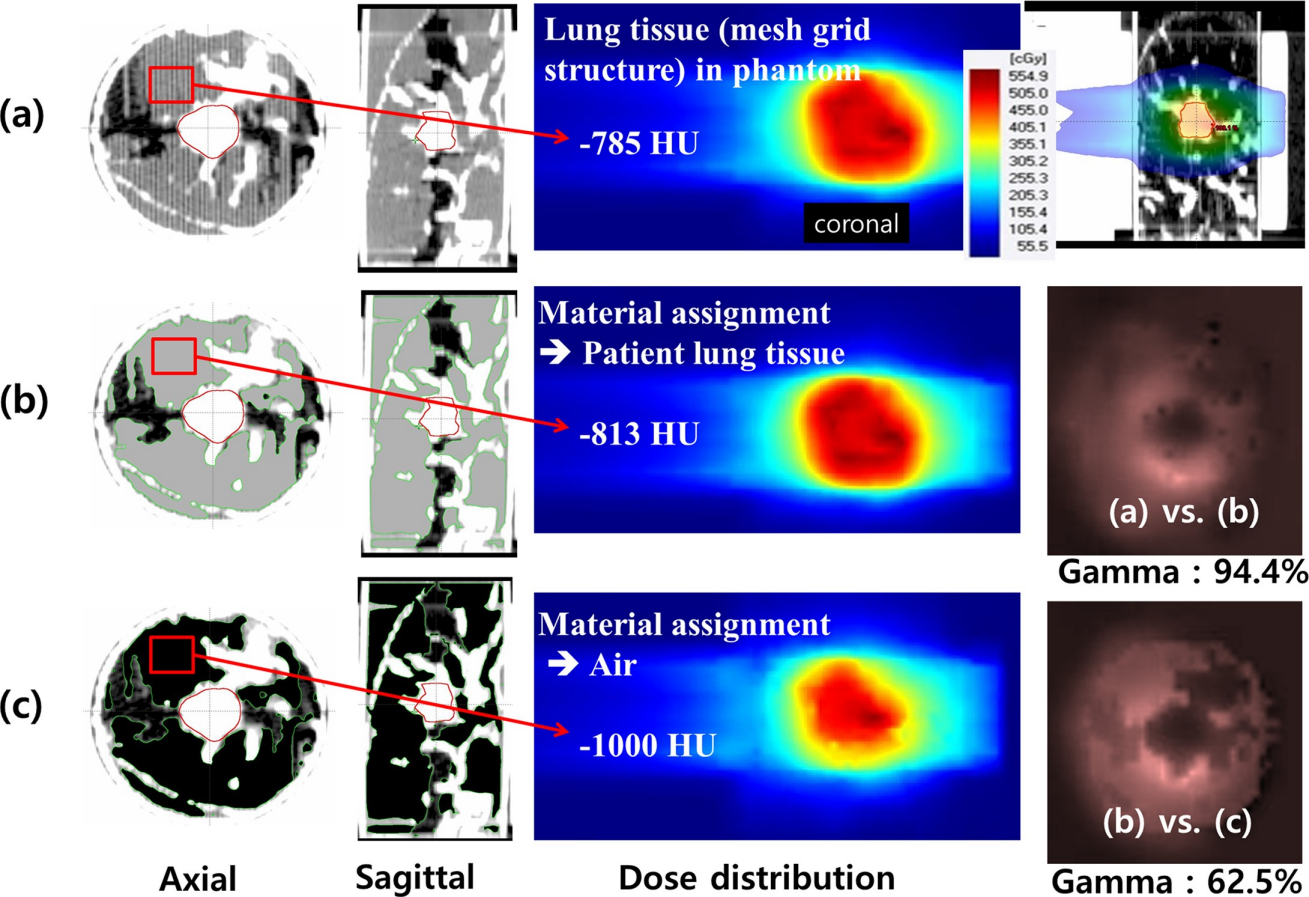
Dosimetric evaluation of the phantoms with lung density reassignment. (a) Axial and sagittal images of the lung phantom are shown. The phantom lung regions were implemented with a mesh structure with 2 mm spacing. The plan dose of 2 arc VMAT is shown in the coronal plane. (b) The CT number of the phantom LTV was replaced by the average lung CT number in the patient’s image (-813 HU). The dose distributions based on the original and reassigned images were compared in the gamma index map. (c) For the HU reassignment with air (-1000 HU), the GPR was 62.5%.

By fusing CT images of the patients and those of their corresponding phantoms as shown in Fig 6, the morphologic agreements for all cases were observed to be good. For evaluating the dosimetric properties of the patient-specific phantoms, the HU reassignments with CT numbers of air (-1000), of water (0) and of patient's lung in the LTV region were carried out. In Fig 7(A), the 2D dose distribution (a) for the double arc VMAT plan calculated via AXB algorithm was compared with the TPS doses based on the reassigned CT images as shown in Fig 7(B) and 7(C) through the gamma index analysis of 3%/0 mm criteria. The GPR for the dose based on the uncorrected CT was observed to be 94.4% in the reassignment with -813 HU, while the GPR was 62.5% in the case of CT number reassignment with -1000 HU of air, as shown in Fig 7. The average CT radiodensity in the LTV of the patients was -813 HU, while the average in the phantom LTV was -785 HU.

For all cases summarized in Table 2, the average GPR with 3%/0 mm criteria was 99.04 ± 2.28% between the original phantom dose and the dose based on the images reassigned with patient-specific LTV CT numbers, while the average GPRs were 74.67% ± 14.07% for the air and 86.30% ± 11.18% for the water CT number reassignments. While the differences in CT number between the patients and the phantoms (mean ΔHU of GTV = 12.7 and mean ΔHU of LTV = 22.3) were observed, the dosimetric influences of those differences were not significant as evidenced by GPRs between patients and phantoms in Table 2.

**Table 2:** HU value of GTV and LTV for six cases and the GPRs (3%/0 mm) compared under various material assignments of LTV in the lung phantoms.

No.	HU of GTV			HU of LTV			Gamma passing rates (3%/0 mm)		
	Patient	Phantom	^a^ΔHU	Patient	Phantom	^a^ΔHU	^c^Patient vs. Air	^d^Patient vs. Phantom	^e^Patient vs. Water
**P1**	78	92	14	-821	-784	37	59.24	99.82	80.77
**P2**	34	53	19	-813	-785	28	62.47	94.39	78.68
**P3**	49	55	6	-794	-801	-7	64.09	100	70.71
**P4**	53	65	12	-717	-708	9	88.83	100	99.28
**P5**	36	46	10	-788	-757	31	86.46	100	94.19
**P6**	33	48	15	-818	-782	36	86.95	100	94.19
**Mean**	47.2	59.8	12.7	-791.8	-769.5	22.3	74.67	99.04	86.30
^**b**^**SD**	17.2	17.1	4.5	39.0	33.3	17.6	14.07	2.28	11.18

^a^ΔHU: HU difference between patient CT image and lung phantom CT image

^b^SD, Standard deviation

^c^Patient vs. Air, = GPR between phantom doses, with the reassignments of the original patient and of air CT number

^d^Patient vs. Phantom = GPR between phantom doses, with and without the reassignments of the original patient

^e^Patient vs. Water = GPR between phantom doses, with the reassignments of the original patient and of water CT number

**Fig 8:**
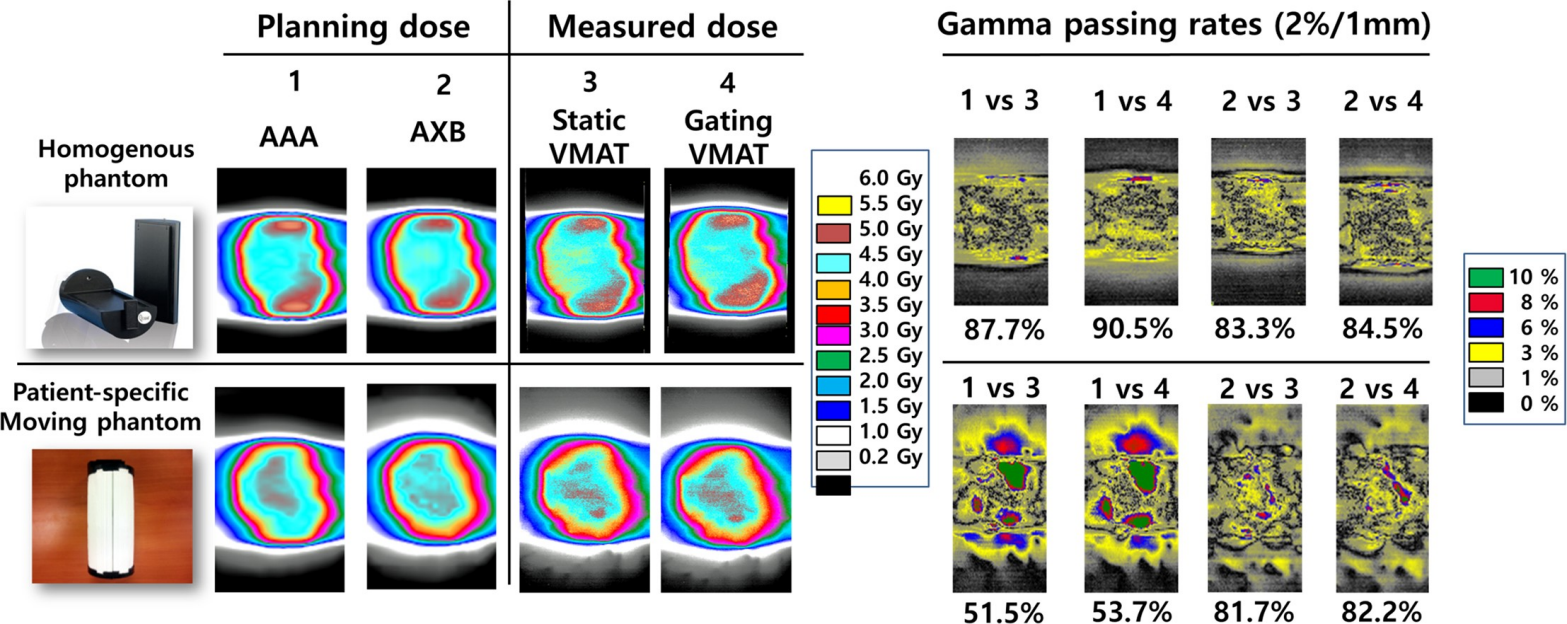
The analysis of the gamma passing rates (2%/1 mm) between the calculated dose and the measured dose comparing VMAT techniques under the static and gating conditions using both homogeneous and inhomogeneous phantom (3D-printed patient-specific moving phantom) in the P2 case. The treatment planning dose was applied using the AAA or the AXB dose calculation algorithm.

In this study, the two types of phantoms (6 heterogeneous and 1 homogeneous) were employed under the two different VMAT delivery conditions (static and gating) and the two types of dose calculation algorithms (AAA and AXB). Here, the static condition indicated that phantoms were stationary without any beam delivery interruption, and the gating condition meant that phantoms were moving in patient-specific patterns during gated beam delivery. As shown in Fig 8, 2 GPRs were generated from the film measurements under the static and gating conditions for one plan dose, while 2 GPRs were generated for the plan doses calculated by AAA and AXB algorithms for one film measurement.

Table 3 shows the GPRs of 2%/1 mm criteria between the calculated plan dose and measured film dose for the influences of respiratory target motion and the lung tissue heterogeneity, using six heterogeneous and one homogeneous phantom.

**Table 3:** The gamma analysis (2%/1 mm criteria) results of the homogeneous and inhomogeneous phantoms (patient-specific lung phantom) for the six cases.

		Homogeneous			Inhomogeneous		
		AAA	AXB	^d^*p*-value	AAA	AXB	^d^*p*-value
**P1**	^**a**^**Static**	**83.2**	**81.4**	**0.094**	**54.7**	**80.2**	0.031*
**P2**		**87.7**	**83.3**		**51.5**	**81.7**	
**P3**		**86.1**	**86.4**		**60.1**	**84.3**	
**P4**		**89.1**	**81.3**		**81.6**	**81.9**	
**P5**		**85.3**	**84.0**		**79.8**	**80.9**	
**P6**		**91.6**	**91.4**		**83.8**	**86.0**	
**P1**	^**b**^**Gating**	**90.5**	**90.4**	**0.438**	**47.5**	**86.8**	0.031*
**P2**		**90.5**	**84.5**		**53.7**	**82.2**	
**P3**		**88.0**	**87.4**		**58.1**	**75.8**	
**P4**		**88.8**	**89.7**		**75.2**	**78.2**	
**P5**		**83.0**	**83.3**		**71.8**	**73.1**	
**P6**		**87.8**	**85.4**		**78.9**	**86.3**	
	^**c**^***p*-value**	**0.844**	**0.313**		**0.094**	**0.563**	
**Mean**	^**a**^**Static and** ^**b**^**Gating**	**87.6**	**85.7**		**66.4**	**81.5**	
^**e**^**SD**		**2.8**	**3.4**		**13.4**	**4.2**	

^a^Static: VMAT plan delivered under the static condition (non-tumor motion and non-gated delivery)

^b^Gating: VMAT plan delivered under the gating condition (tumor motion and gated delivery)

^c^ *p*-value between GPRs of Static and of Gating conditions for each algorithm

^d^ *p*-value between GPRs of AAA and of AXB conditions

^e^SD, Standard deviation

*Values significant with respect to a *p*-value of 0.05.

Regarding the condition of beam delivery, no significant differences in GPR were observed (homogeneous phantom, *p* = 0.844 for AAA and 0.313 for AXB; inhomogeneous phantom, *p* = 0.094 for AAA and 0.563 for AXB). On the other hand, there were significant differences between GPRs of the AAA and AXB algorithms for inhomogeneous phantoms (*p* = 0.031 for both static and gating conditions).

## Discussion

To validate the implementation of lung tissue in the patient-specific phantom, the TPS-calculated doses and HU values of both the lung tissue and tumor of each patient were compared to values of the lung phantoms. Although the EBT film sheet of 0.2 mm thick water equivalent materials could provide the influence of the dosimetric measurements in air, any impacts of film measurements would hardly be expected for the lung phantoms with mesh structures formed as the 40 plastic sheets of 0.3 mm thickness. By the plan dose comparisons in various CT number reassignments, the patient-specific lung phantoms were dosimetrically verified for utilization in this study.

As the consideration of the low sample size would be critical in studying the 6 enrolled cases, all measurements under possible conditions of the homogeneity of phantoms, dose calculation algorithms, and beam delivery conditions were performed. Through the independent measurements for the 6 enrolled cases under various conditions, 48 GPRs could be generated. As summarized in Table 3, GPRs of the static and gating conditions were not significant regardless of the kinds of enrolled phantoms (p > 0.05). This means that the protocol of beam delivery with 30–70% phase gating was well operated for the breathing patterns of the six patients shown in Fig 4.

The GPRs between calculation algorithms were statistically significant for heterogeneous (*p* = 0.031) phantoms in both static and gating conditions. The statistical significance between algorithms under heterogeneous condition reflects the known fact that the doses calculated using the AAA algorithm would not be proper for lung SBRT cases. These results were consistent with the previously reported studies of the AXB algorithm, which has been reported to show improved dose accuracy compared to the AAA algorithm in heterogeneous media and near interfaces of different material densities [23–26].

Under the realistic conditions of the gating delivery for target motions and the heterogeneous environments for VMAT, among 6 GPRs for AXB calculation, 50% passed and the other 50% of cases were marginally lower than the acceptance threshold 80%, while all GPRs of AAA failed. For the conventional QA condition of static delivery and homogenous conditions, GPRs were all acceptable for both AAA and AXB calculations. For dosimetric verification of gated VMAT for lung cancer, both dose calculation accuracy and patient breathing pattern should be considered due to the heterogeneous media of lung tissue with tumor motion. However, the conventional method has not generally reflected these issues.

In detail on the variation of case by case, the GPR differences between the algorithms for the case of P1, P2, and P3 were more than 20 with the stationary condition for the heterogeneous phantoms, while the others were less than 5. Also, when the verification of lung phantom fabrication qualities were examined by the HU reassignment with air value, the reductions in GPR for these three cases were much greater than the others. All the above occurred since in relative comparison with other cases, these three phantoms were presented to have tumor volume smaller than 10 cm^3^, as well as LTVs larger than 250 cm^3^. Those results well reflected that the larger disequilibrium of charged particles caused by the more heterogeneous condition enlarged the GPR differences between dose calculation algorithms.

This study has some limitations. Organ deformations in accordance with target movements could not be implemented with the 3D-printed phantom and only one-dimensional movement in the superior and inferior directions was available to simulate the respiratory organ movements using the motion device. Furthermore, the experiment involved only 6 cases, as printing individual phantoms is time and resource-intensive. On average, a single phantom fabrication takes about 24 hours. This 3D printing process might not be practical for the general patient-specific QA. However, for the end-to-end verification of new treatment techniques like this study, it is pretty certain that there is a lot of benefit in the clinical adaptation phase.

## Conclusions

The usefulness of heterogeneous patient-specific lung phantoms moving in actual breathing patterns was evaluated to be essential in such verification processes of gated VMAT delivery. In addition, it was dosimetrically confirmed that the AXB algorithm improved the dose calculation accuracy under patient-specific simulations using 3D printed lung phantom. The most critical factor which affected the accuracy of gated VMAT in lung SBRT was determined to be the dose calculation algorithm accuracy rather than target movements in the enrolled cases for this study.

## Supporting information

S1 Table The entire GPR data set with 3%/3 mm, 2%/2mm and 2%/1mm criteria of the homogeneous and inhomogeneous phantoms (patient-specific lung phantom) for the six cases. Abbreviations: GPR: gamma passing rates.
(PDF)
