# Supplementary material for: Dosimetric evaluation of respiratory gated volumetric modulated arc therapy for lung stereotactic body radiation therapy using 3D printing technology
Source: PLoS One. 2018 Dec 26;13(12):e0208685. doi: 10.1371/journal.pone.0208685 (PMC6306268; doi:10.1371/journal.pone.0208685)
Supplement: S1 Table — Abbreviations: GPR: gamma passing rates. (PDF) [file pone.0208685.s001.pdf]

GPR 3%/3mm	1	2	3	4	5	6	7	8
	Homogeneous				Inhomogeneous			
	AAA		AXB		AAA		AXB	
	static	gating	static	gating	static	gating	static	gating
P1	99.80	99.60	99.60	99.50	95.30	87.80	98.80	98.80
P2	99.30	98.60	99.40	98.60	94.20	85.30	99.10	98.10
P3	99.20	98.50	99.20	98.30	95.10	86.20	99.30	97.80
P4	99.10	98.40	99.10	98.50	99.10	98.80	99.20	98.90
P5	99.20	98.70	99.20	98.60	98.50	97.10	98.80	98.50
P6	99.30	99.10	99.30	99.10	98.80	98.80	99.10	99.10
Mean	99.32	98.82	99.30	98.77	96.83	92.33	99.05	98.53
SD	0.25	0.45	0.18	0.45	2.19	6.54	0.21	0.50

GPR 2%/2mm	1	2	3	4	5	6	7	8
	Homogeneous				Inhomogeneous			
	AAA		AXB		AAA		AXB	
	static	gating	static	gating	static	gating	static	gating
P1	95.75	99.09	95.09	99.21	78.93	68.81	94.44	98.50
P2	97.02	96.63	94.70	95.63	72.27	75.11	93.27	93.80
P3	95.77	97.51	95.69	97.51	80.84	74.74	95.45	90.93
P4	96.42	96.22	93.30	97.10	96.19	90.54	96.03	95.35
P5	94.81	96.49	95.51	96.02	94.53	89.47	94.64	91.23
P6	96.74	97.55	96.75	96.25	94.77	92.27	97.07	97.06
Mean	96.09	97.25	95.17	96.95	86.26	81.82	95.15	94.48
SD	0.81	1.06	1.15	1.31	10.18	10.08	1.33	3.07

GPR 2%/1mm	1	2	3	4	5	6	7	8
	Homogeneous				Inhomogeneous			
	AAA		AXB		AAA		AXB	
	static	gating	static	gating	static	gating	static	gating
P1	83.2	90.5	81.4	90.4	54.7	47.5	80.2	86.8
P2	87.7	90.5	83.3	84.5	51.5	53.7	81.7	82.2
P3	86.1	88.0	86.4	87.4	60.1	58.1	84.3	75.8
P4	89.1	88.8	81.3	89.7	81.6	75.2	81.9	78.2
P5	85.3	83.0	84.0	83.3	79.8	71.8	80.9	73.1
P6	91.6	87.8	91.4	85.4	83.8	78.9	86.0	86.3
Mean	87.2	88.1	84.6	86.8	68.6	64.2	82.5	80.4
SD	3.0	2.8	3.8	2.9	14.7	12.8	2.2	5.6
